# Postprandial Metabolite and Antioxidant Kinetics Following Intake of a Carob Beverage in Healthy Males

**DOI:** 10.3390/nu18132190

**Published:** 2026-07-05

**Authors:** Stamatia-Angeliki Kleftaki, Thalia Tsiaka, Charalampia Amerikanou, Demetra Sigala, Aikaterini Mavroudi, Maria-Myrto Karagiorgou, Altenisa Kuci, Chara Tzavara, Vasiliki Dima, Maria Morfiadaki, Aristea Gioxari, Panagiotis Zoumpoulakis, Andriana C. Kaliora

**Affiliations:** 1 Department of Nutrition and Dietetics, School of Health Science and Education, Harokopio University of Athens, 70 El. Venizelou Ave., 17676 Athens, Greece; matina.kleftaki@gmail.com (S.-A.K.); amerikanou@windowslive.com (C.A.); sigalademetra@gmail.com (D.S.); caterina@mavroudis.gr (A.M.); marymkar@gmail.com (M.-M.K.); koutsialtenisa@gmail.com (A.K.); htzavara@med.uoa.gr (C.T.); a.gioxari@go.uop.gr (A.G.); 2 Department of Food Science and Technology, University of West Attica, Agiou Spyridonos, 12243 Egaleo, Greece; tsiakath@uniwa.gr (T.T.); fst20684149@uniwa.gr (V.D.); fst19684059@uniwa.gr (M.M.); pzoump@uniwa.gr (P.Z.)

**Keywords:** carob, postprandial kinetics, gut-derived metabolites, antioxidant capacity

## Abstract

**Background/Objectives:** *Ceratonia siliqua* L. (carob) is a rich source of bioactive compounds with potential health-promoting properties. This study investigated the kinetics of serum metabolites following the consumption of a carob beverage and evaluated associated changes in circulating antioxidant status. **Methods:** Fifteen apparently healthy adult men completed an acute postprandial intervention; only male participants were included to minimize the biological variability related to sex-dependent differences in phytochemical kinetics and antioxidant responses. Participants consumed a beverage from carob pod powder (30 g) dispersed in water (200 mL). Blood samples were collected at baseline and every 30 min for 6 h following intake. Serum metabolic profiling was performed using a non-targeted liquid chromatography–time-of-flight mass spectrometry (LC-TOF-MS) approach. Antioxidant responses were assessed by measuring ferric-reducing antioxidant power (FRAP) and serum resistance to copper sulphate-induced oxidation. **Results:** Twenty-four putative metabolites were detected, including phenolic compounds, fatty acids, amino acids, dipeptides, monosaccharides, pyridoxine, and gut microbiota-derived metabolites. Urolithin B appeared at 30 min (28.0 ± 4.0 × 10^2^ a.u.), while p-cresol sulfate increased from 53.3 ± 6.5 × 10^2^ a.u. at baseline to 130.0 ± 7.0 × 10^2^ a.u. at 30 min. FRAP values did not change significantly over time (*p* = 0.332), whereas oxidation lag time showed a significant time effect (*p* = 0.001), reaching its highest mean at 180 min (9093.5 ± 1885.1 s). **Conclusions:** Carob beverage consumption resulted in a diverse postprandial serum metabolite profile. Antioxidant responses appeared to be only partly explained by circulating phenolics, suggesting that additional pathways and bioactive constituents may contribute.

## 1. Introduction

*Ceratonia siliqua* L. (Fabaceae: Caesalpinioideae) (carob) is an evergreen Mediterranean tree (Fabaceae) 8–15 m tall, with thick, glossy pinnate leaves, autumn-flowering racemes of small apetalous flowers, and indehiscent pods (10–30 cm) containing sweet pulp and hard seeds. Carob is considered to be rich in polyphenols and other bioactive compounds. Several methods have been applied in pods to determine the polyphenol content, and most of them reveal the presence of phenolic acids, such as gallic acid, condensed tannins formed by flavan-3-ols, and gallate esters, such as catechin, epicatechin, and epigallocatechin gallate, and flavonoids [1,2,3]. Previous studies have demonstrated antioxidant and other biological activities of *Ceratonia siliqua* extracts, largely attributed to their phytochemical composition [4]. The high phenolic content has been associated with several antioxidant activities in Carob extracts in vitro, such as high free-radical-scavenging activity [3], decrease in reactive oxygen species (ROS) levels [5] and several other health benefits, such as hypolipidemic, antidiabetic, hepatoprotective and other activities [6].

Beyond its phytochemical composition, carob represents an agronomically and economically relevant Mediterranean crop. The tree is well adapted to semi-arid environments, low water availability, and marginal soils, making it particularly suitable for cultivation under changing climatic conditions [7]. Carob pods are used for food applications, while the seeds are an important source of locust bean gum, a valuable ingredient for the food industry [7]. Despite this potential, carob remains relatively underutilized compared with other Mediterranean crops, and further valorization of local cultivars is needed. In this context, the Cretan “Imera” cultivar is of particular interest, as it has been previously characterized by our group for its phytochemical profile and glycemic regulatory effects [8]. Among the polyphenols identified in the Cretan carob cultivar “Imera” are gallic acid, catechin, quercetin, myricetin, and kaempferol [8].

Several in vitro digestibility and bioaccessibility studies have been performed on pods from carob. Ait Chait and his colleagues conducted an in vitro simulation of human digestion of pulp and showed that the gastric phase reduced the phenolic and flavonoid content, whereas soluble free phenolics and flavonoids were increased during intestinal digestion, suggesting that intestinal enzymes and bile salts facilitate the release of bound phenolics [9,10]. In contrast, free phenolic compounds were shown to decrease under gastrointestinal conditions in carob flour, possibly due to the influence of dietary fiber and its interactions with polyphenols [11].

Although carob phenolics and antioxidant capacity have been widely studied in vitro, their in vivo absorption and metabolic fate remain poorly characterized. The occurrence of phenolic compounds in human biological samples is usually studied with targeted analyses that allow for the identification and quantification of targeted metabolites. However, targeted approaches may fail to provide a more comprehensive profile of human metabolites. Consequently, non-targeted metabolite screening approaches are preferred, as they can reveal metabolites that were not preselected, including novel or unexpected compounds [12].

We hypothesized that a non-targeted LC-TTOF-MS-based approach, combined with the assessment of postprandial antioxidant kinetics, would help explore the metabolic impact of carob consumption. Therefore, we conducted an open-label, single-group, postprandial kinetic metabolite study where participants consumed a carob-based beverage. To ensure clinical relevance, the Cretan carob cultivar “Imera” was selected, previously characterized by our group for its glycemic regulatory effects and rich phytochemical profile [8]. Our aim was to (a) characterize carob-derived bioactive compounds and endogenous blood metabolites in serum samples and (b) identify the time-dependent alterations of the metabolites (primary endpoint) and antioxidant capacity (secondary endpoint) related to the consumption of a carob-based beverage.

This design provides a first insight into carob’s systemic effects and enables a comprehensive assessment of its potential role in functional nutrition and metabolic health. This exploratory work serves as a “proof-of-concept” time-course analysis, mapping for the first time the dynamic postprandial appearance of metabolites, either carob-derived or not, in human circulation.

## 2. Materials and Methods

### 2.1. Plant Material

Plant material of *Ceratonia siliqua* L., specifically, the pods, was collected from the carob trees of the Imera cultivar in the village Pines in Elounda, Prefecture of Lasithi (coordinates L:35.278545, A:25.715529). The tree was botanically identified by Professor Kalantidis, Department of Biology, University of Crete, Greece. The resulting powder was packaged in airtight containers and was used for the study. Chemical profiling (LC-TTOF MS analysis) revealed a characteristic profile of carbohydrates and sugars, including d-pinitol, structural disaccharides and monosaccharides, organic acids, flavonoids, isoflavonoids, and phenolic compounds (primarily driven by high-intensity compounds like gallic acid, catechin, and myricetin), alongside diverse flavonoid glycosides [8].

### 2.2. Ethics and Trial Registration

This is an open-label, single-group, acute postprandial kinetic study in apparently healthy participants, conducted at Harokopio University of Athens. The study protocol was reviewed and approved by the Harokopio University Ethics Committee (43-18/01/2023) and was carried out in accordance with the principles of the Declaration of Helsinki and Good Clinical Practice guidelines. The trial was registered at ClinicalTrials.gov (Identifier: NCT05870930, 12 May 2023). Participants received free blood tests, anthropometric assessments, including body composition analysis, and dietary counseling as compensation for their time. However, no monetary remuneration was provided. The study complied with all applicable international and national laws, regulations and guidelines on the conduct of clinical trials. Prior to enrolment, participants were provided with a comprehensive information leaflet detailing the study’s objectives, methodology, protocol and potential benefits. They were instructed to read this carefully before signing two copies of the informed consent form, retaining one for their records. Participants were explicitly informed of their right to withdraw from the study at any time.

### 2.3. Participants

**Figure 1:**
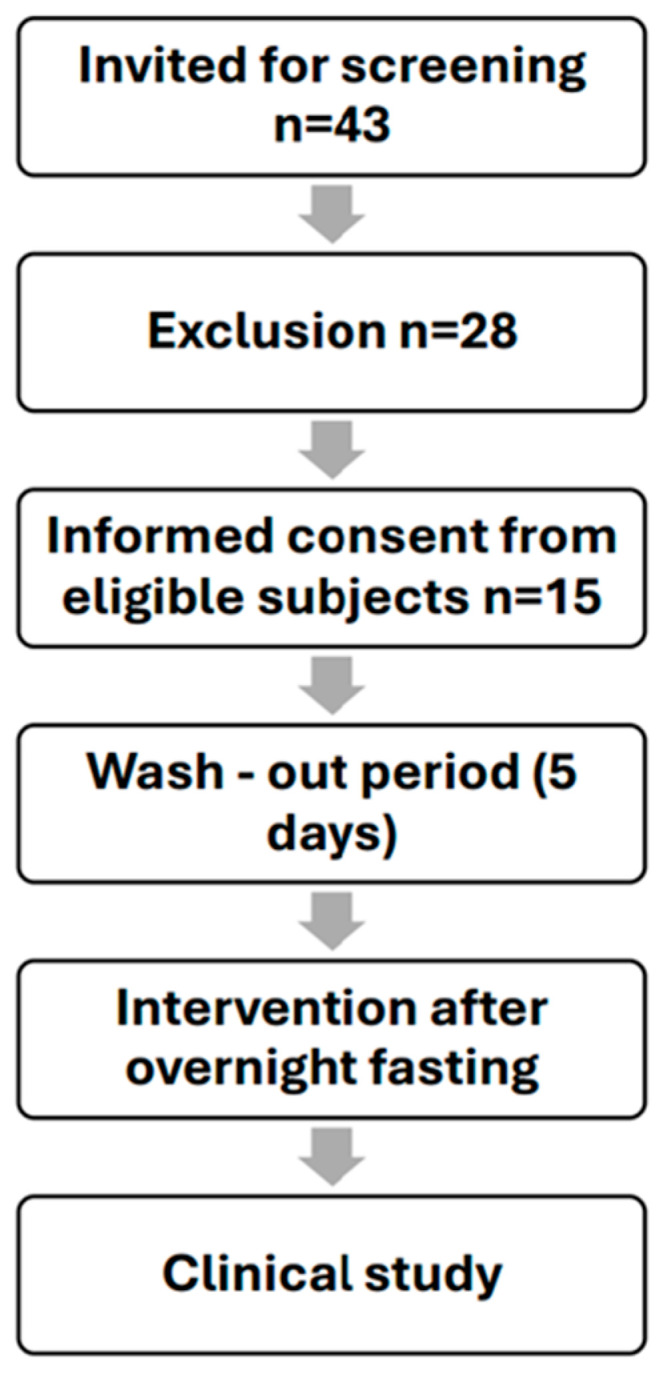
Study flowchart.

Forty-three apparently healthy male adults were initially invited for screening (Figure 1). Male sex was selected as an inclusion criterion in order to minimize the confounding effect of hormonal fluctuations, sex-related body composition, and protein-binding differences that may influence bioavailability and the distribution of compounds. Based on the inclusion and exclusion criteria (Table 1), fifteen volunteers were enrolled. The sample size was determined based on our previous postprandial intervention study using the same carob-based beverage, in which significant effects on postprandial glycemic responses were detected in healthy individuals. Considering the within-subject time assessment and the exploratory nature of postprandial metabolic profiling, a cohort of 15 participants was sufficient to capture the relevant changes in circulating metabolites and kinetic antioxidant potential following consumption of a carob beverage (carob pod powder dispersed in water). Recruitment took place from February to October 2024 via poster notifications.

### 2.4. Postprandial Study Protocol

After enrollment, participants underwent a comprehensive medical and dietary assessment. Anthropometric measurements were obtained. Body weight was measured to the nearest 0.1 kg using a calibrated scale. Body composition (% fat, free fat mass, and total body water) was assessed by bioelectrical impedance analysis (Tanita BC-418, Tokyo, Japan). Waist circumference (WC) was measured at the midpoint between the lower edge of the last palpable rib and the apex of the iliac crest, with participants standing and following a gentle exhalation. Height was recorded to the nearest centimeter using a calibrated stadiometer (Seca 217, Seca, Hamburg, Germany). Body mass index (BMI) was calculated by dividing body weight (kg) by the square of height (m^2^). Participants were classified as underweight, normal weight, overweight or obese according to World Health Organisation (WHO) BMI criteria.

Prior to the intervention, participants were given an indicative nutritional plan to adhere to a low-phytochemical diet for five consecutive days (Table 2), which excluded major dietary sources of phytochemicals such as fruits, vegetables, legumes, coffee, tea, alcoholic beverages, and chocolate. Compliance with the low-phytochemical diet was assessed by 24 h dietary recalls conducted during and at the end of the washout period. Following an overnight 12 h fast, the participants reported to the Metabolism Unit of Harokopio University for anthropometric and body-composition reassessment.

**Figure 2:**
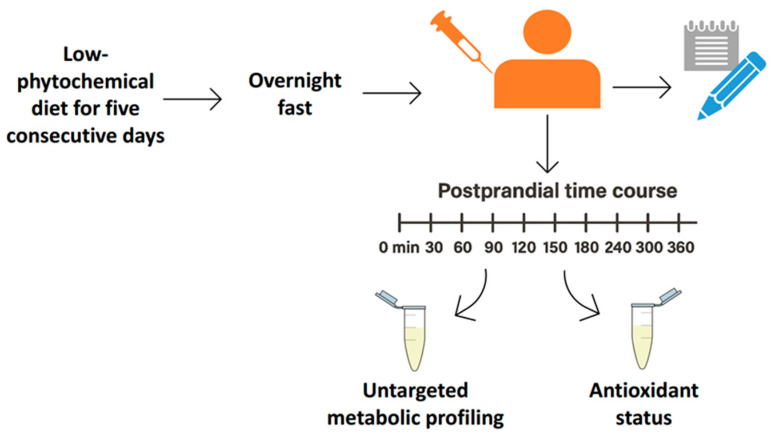
Blood-sampling procedure.

Volunteers consumed a carob beverage prepared by dispersing 30 g of powder in 200 mL of drinking water. Hence, the total phenols and phytochemical profile were considered equivalent to those previously reported. A venous catheter was placed, and a 20 mL blood sample was collected for antioxidant capacity and kinetic analyses. Blood samples were drawn at baseline (0 min) and at 30, 60, 90, 120, 150, 180, 240, 300 and 360 min postprandially. Immediately after collection in serum separator tubes, the samples were centrifuged at 3000 rpm for 10 min at 4 °C to obtain plasma and serum, which were stored at −80 °C until further analyses. The participants were instructed not to consume any food or beverages throughout the postprandial period. Only 200 mL of water was allowed to be consumed ad libitum throughout the 6 h (Figure 2).

### 2.5. Laboratory Analysis

#### 2.5.1. Sample Preparation

For sample preparation, serum samples were thawed gently on ice and vortexed. Then, 200 μL from each sample was mixed with 600 μL of ice-cold acetonitrile (ACN) in order to precipitate and remove serum proteins. Following this, the samples were homogenised as they were vortexed for 30 s and then incubated for 20 min at −20 °C to further facilitate protein precipitation. Thereafter, the serum samples were centrifuged at 12,500 rpm at 4 °C, and the supernatants, containing polar and moderately polar metabolites, were collected. Finally, the solvent (ACN) was removed using a gentle nitrogen stream, and the obtained dried residues were reconstituted in 500 μL of mobile phase (90% H_2_O-10% ACN containing 0.1% formic acid).

Furthermore, representative quality control (QC) samples were prepared by pooling 13.3 μL of each of the 15 samples from each time point (*n* = 15) and by following the same procedure as described above. After reconstitution, all samples were filtered with a Chromafil Xtra PET filter 45/13 (Macherey-Nagel, Düren, Germany) and were ready for injection.

All solvents used for sample preparation and LC-MS analysis were of LC-MS grade. Specifically, water and acetonitrile were obtained from Fisher Chemical (Loughborough, Leicestershire, UK), and formic acid was acquired from Carlo Erba reagents (Milan, Italy).

#### 2.5.2. Micro-Liquid Chromatography Coupled with Triple Quadrupole Time-of-Flight Mass Spectrometer (microLC-TTOF MS) Analysis

The applied microLC-MS method was based on the methods and technical notes of Amerikanou et al. (2025), Kvirencova et al. (2023) and Jiang et al. (2019), with minor modifications [8,13,14]. In more detail, the elution of serum metabolites was performed on an Ekspert nanoLC 425 system (Eksigent^®^, Dublin, CA, USA) equipped with a Phenomenex Luna C-18 (2) (100 Å, 150 mm × 0.3 mm, 3 μm particle size; Phenomenex, Torrance, CA, USA) reversed-phase (RP) column. The gradient elution program consisted of solvent A (water with 0.1% (*v*/*v*) formic acid) and solvent B (acetonitrile with 0.1% (*v*/*v*) formic acid) at a 10 µL/min flow rate. The gradient profile was run as follows: t = 0–1 min, 10% B; t = 1–6 min, linear increase from 10% to 90% B; t = 6–7.5 min, 80% B; t = 7.5–7.6 min, decrease from 80% to 20% B; and t = 7.6–13 min, return to the initial condition (10% B) for column re-equilibration. The sample injection volume was 1 µL for both the serum and blank samples, while the column oven temperature was set at 35 °C. The retention time variation among QC injections remained below ±0.2 min.

Mass spectrometric analysis was conducted with the triple-quadrupole time-of-flight mass spectrometer TripleTOF^®^ 6600+ (Sciex, Redwood City, CA, USA) operating in both positive and negative ionisation modes, while the column eluent was inserted directly into the mass spectrometer by electrospray ionisation (ESI) source. Data acquisition was carried out in information-dependent acquisition (IDA) mode to enable MS2 fragmentation of the precursor ions. The ion spray voltage was adjusted to −4500 V and the source temperature to 300 °C. The curtain gas (CUR), ion source gas 1 (GAS1) and ion source gas 2 (GAS2) were set at 30, 40, 40 a.u, respectively, while the declustering potential (DP) was equal to 80 V. The collision energy (CE) was set at 10 V for the MS scans and 35 (±5) V for the MS/MS experiments, since higher energy is required for molecular ions’ fragmentation. For the mass acquisition, a total of 11,311 cycles was recorded, with a duration of 0.5950 s per cycle. The mass scan range was delimited at *m*/*z* = 100–1000 and *m*/*z* = 80–700 for the MS and MS/MS experiments, individually, and the mass tolerance was set to 10 ppm.

The LC-MS instrumentation was controlled using Analyst^®^ TF Software 1.8 (Sciex, CA, USA).

#### 2.5.3. Data Processing

The obtained mass spectra were processed using Sciex OS Q (Sciex, CA, USA).

Putative identification of metabolites based on monoisotopic masses was performed by applying a suspect list of carob-derived [7,15,16,17] and serum metabolites [1,8,18,19,20,21,22,23] compiled from reported data in combination with reference spectra from the Human Metabolome Database (HMDB, https://www.hmdb.ca/ (accessed on 28 June 2026)) and Mass Bank (https://massbank.eu/MassBank/ (accessed on 28 June 2026)). Namely, the criteria for compound identification were (a) precursor ion *m*/*z* accuracy within the ±10 ppm value compared to the reported mass and/or (b) matching of at least two common fragment ions (MS/MS) with entries from the suspect list and HMDB and/or Mass Bank reference spectra. Moreover, the spectra quality was adjusted adequately to exclude precursor ions with poor spectrum quality in order to minimize false-positive identifications, which may be attributed to baseline fluctuations or noise inherent to the instrument.

### 2.6. Antioxidant Capacity Assays

As part of the 6 h sampling protocol, kinetics in the antioxidant potential were recorded for the samples collected up to 180 min to capture the rapid postprandial phase in which antioxidant changes are most pronounced.

The ferric-reducing antioxidant power (FRAP) assay is based on the ability of serum to reduce ferric (Fe^3+^) to ferrous (Fe^2+^), based on the electron-donating action of antioxidants that are present in serum. We used the FRAP Assay Kit (colorimetric, ab234626, Abcam, Waltham, MA, USA), according to the manufacturer’s instructions. Briefly, 10 μL of serum samples or ferrous standards (0, 4, 8, 12, 16 and 20 nmol/well) were combined with a 190 μL reaction mix (FRAP Assay Buffer, FRAP Probe, and Ferric Iron Solution (Abcam, Waltham, MA, USA), incubated for 60 min at 37 °C, and absorbance was measured at 594 nm. Sample concentrations were calculated from the corresponding standard calibration curve (R^2^ = 0.9993, y = 0.054x − 0.0013) and expressed as nmol ferrous equivalents. In addition to the chemical-based FRAP assay, we measured the lag time preceding serum oxidation, a more physiologically relevant assay that provides a kinetic assessment of the serum endogenous protection against oxidative damage to its lipid fraction [21]. Serum was diluted with phosphate-buffered saline and was placed in a flat-bottomed plate, where copper sulfate (CuSO4) solution was added to promote oxidation. Oxidation kinetics were monitored for 3 h, as the total serum lipoproteins were oxidized in vitro by copper sulphate. Conjugated dienic hydroperoxides that were produced were measured at 245 nm, and the absorbance was plotted against time. The data are expressed as lag time preceding oxidation and are expressed in seconds.

All samples were measured in duplicate, and absorbance was measured in an ELISA reader ((PowerWave XS2, BioTek Instruments, Winooski, VT, USA).

### 2.7. Statistical Analysis

Mean and SD apply to quantitative variables. Mean antioxidant capacity levels were compared between time points using a repeated measures analysis of variance (ANOVA), applying Bonferroni correction for multiple comparisons. Data were analysed using the Statistical Package for Social Sciences (SPSS 21.0, SPSS Inc., Chicago, IL, USA), and the level of statistical significance was set at *p* < 0.05.

The statistical analysis and visualization of the LC-MS results were carried out with Minitab (20.1, Minitab LLC, State College, PA, USA). Differences between the average intensities of the examined samples at each time point were assessed by one-way ANOVA at a confidence level of 95%, followed by a Tukey post hoc test.

## 3. Results

Fifteen subjects met the inclusion criteria tightly and completed the study. Table 3 presents their baseline characteristics. The mean (SD) age of the participants was 24.47 (4.82) years. The mean (SD) of the anthropometric characteristics confirmed that the population was of normal weight (weight: 76.85 ± 6.63 kg, BMI: 24.65 ± 1.79 kg/m^2^, % fat: 15.35 ± 3.87, free fat mass: 64.96 ± 5.15 kg). Systolic and diastolic pressure and heart rate were within the normal reference range.

**Figure 3:**
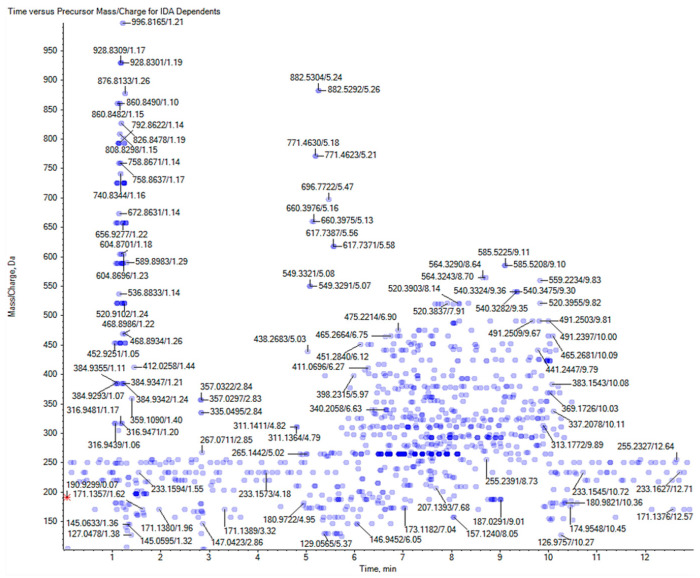
Metabolite map of QC30 at negative ionization. Each dot represents the precursor ion (y-axis) of a compound eluted at specific retention time (x-axis). Darker blue coloring corresponds to higher intensities of the respective metabolite in the sample. The asterisk indicates a precursor ion selected in SCIEX OS for MS/MS inspection.

Based on the criteria described above (Section 2.4), 24 tentative metabolites were identified, including parent compounds, whose presence in serum could be ascribed to the consumption of the carob beverage, since they were also reported in this substrate [8] and endogenous metabolites, whose precursor mass intensities, and therefore their levels, could be affected by carob intake. A mass map (*m*/*z* of precursor ion vs retention time) of serum metabolites recorded, indicatively, in a QC sample at t = 30 min (QC30) in negative ionization is presented in Figure 3, while the MS/MS fragments of the selected annotated metabolites are illustrated in Figure S1a–d (Supplementary Data).

**Figure 4:**
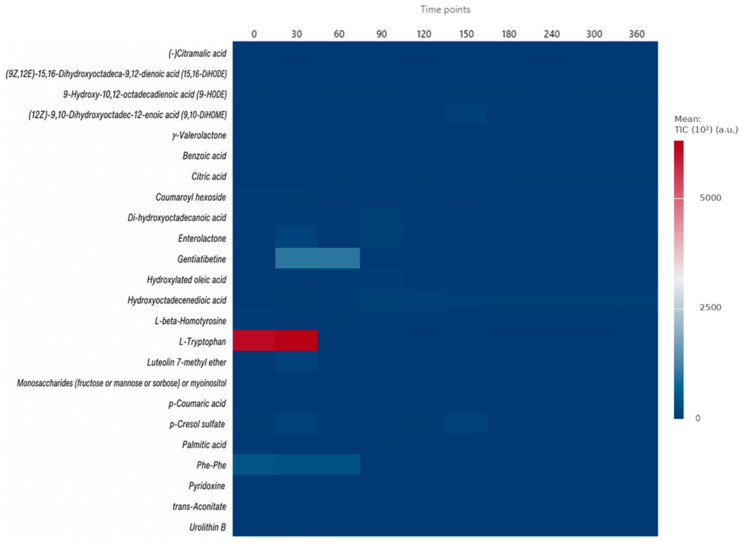
Time-resolved heatmap of metabolite intensity changes during different time points. Color intensity represents the mean total ion chromatogram (TIC) peak intensity, expressed as ×10^2^ arbitrary units (a.u.), with darker blue indicating lower intensity and red indicating higher intensity.

Time-resolved changes were depicted in a heatmap graph (Figure 4), while Table 4 summarizes the annotation characteristics of the putative metabolites and the statistical information regarding their temporal occurrence across the different time points.

The majority of the elucidated metabolites fall within the categories of phenolic compounds (i.e., phenolic acids, flavonoids, etc.), fatty acids, and amino acids, whereas three of the detected compounds (enterolactone, urolithin B, and γ-valerolactone) were gut-derived products formed by microbial metabolism of phenolic constituents. More specifically, (−) citramalic acid, luteolin 7-methyl ether, trans-aconitate and urolithin B were present only 30 min after the carob beverage consumption. Benzoic acid, pyridoxine and p-coumaric acid were present only 60 min and 9,10-DiHOME only 150 min after the carob beverage consumption. Gentiatibetine, monosaccharides and myoinositol were present 30 and 60 min, hydroxylated oleic acid 60 and 90 min, palmitic acid 30, 90, 150 and 240 min and γ-valerolactone B 180 and 240 min after the carob beverage consumption. 9-HODE was present at all timepoints, and its peak intensity did not change between those. Coumaroyl hexoside and L-tryptophan were present at baseline and at 30 min with no significant difference between the two time points. Citric acid and hydroxyoctadecenedioic acid were constantly present (t = 60 to t = 360 min and t = 30 to t = 360 min, respectively). 15,16-DiHODE was higher at 30, 120 and 240 min after post-consumption. Di-hydroxyoctadecanoic acid and L-beta-homotyrosine were higher at 90 and 60 min, respectively, post-consumption, compared to the remaining. Additionally, enterolactone was higher at 30 and 90 min post-consumption. p-Cresol sulfate exhibited a more than two-fold peak intensity at 30 and 150 min post-consumption. Phe-phe peak intensities were lower 30 and 60 min post-consumption. The fact that a number of metabolites, namely 15,16-DiHODE, 9-HODE, coumaroyl hexoside, di-hydroxyoctadecanoic acid, enterolactone, L-beta-homotyrosine, L-tryptophan, p-Cresol sulfate and phe-phe, exist even at baseline does not exclude the possibility of incomplete washout or endogenous production of these metabolites.

Regarding the kinetics in the serum antioxidant potential, the mean values of antioxidant capacity in each time point, as measured by FRAP assay and total serum oxidisability, are presented in Table 5. Post hoc analysis with least significant difference (LSD) showed significant differences in FRAP at 150 min, with higher values than at 30 and 90 min, while lag time was increased at 30, 90, and 180 min, where it reached its highest mean. However, these differences did not survive strict Bonferroni correction for multiple comparisons, suggesting a high variability across participants.

## 4. Discussion

Hereby, the serum metabolic profile following consumption of a carob beverage from pods of the Imera cultivar (Crete, Greece) was investigated. Additionally, the study assessed the kinetics in serum FRAP levels and serum resistance to oxidation and examined their potential association with the occurrence of compounds and metabolites identified through a microLC-TTOF MS analysis of the post-consumption serum samples. This work complements previously published findings on the phytochemical characterization of these pods and their documented effects on postprandial glycemic responses [8].

Following consumption, several metabolites, including (−) citramalic acid, luteolin 7-methyl ether, trans-aconitate, urolithin b, bensoic acid, pyridoxine, p-coumaric acid, 9,10-DiHOME gentiatibetine, monosaccharides and myoinositol, hydroxylated oleic acid, palmitic acid and γ–valerolactone, were detected only post-intake and were not observed at baseline. (−) Citramalic acid, luteolin 7-methyl ether, trans-aconitate and urolithin B were present early after the consumption, possibly indicating rapid metabolic degradation [24].

Benzoic acid, pyridoxine and p-coumaric acid were also detected following carob beverage consumption. Benzoic acid and p-coumaric acid are small phenolic acids, and their appearance may be possibly due to the degradation of carob polyphenols. Benzoic acid was also present during the phytochemical analysis of the aqueous methanolic extract [7]. Its presence may be consistent with the composition of the pod, which has been reported as a source of B complex vitamins [25].

Citric acid, an intermediate of the tricarboxylic acid (TCA) cycle and an important metabolic regulator, followed a different excretion pattern (60, 120, 180, 240, 300 and 360 min), with no significant differences over time (*p*-value ≥ 0.05). Citric acid has been detected in the methanolic extract [8]. Although citric acid is known to scavenge free radicals, including hydrogen peroxide, hydroxyl, alkoxy, peroxide radicals, and superoxide anions26, its antioxidant potential in the present study remains uncertain, as observed increases in antioxidant capacity did not survive the Bonferroni correction.

Regarding the fatty acids identified in both the preparation and volunteers’ serum, hydroxylated oleic acid was detected. This metabolite might be generated by gut bacteria, participating in the regulation of oxidative stress and inflammation. Furthermore, hydroxyoctadecenedioic acid was detected at all time intervals, with the highest peak intensity observed at 120 min, probably due to its partial metabolism. Di-Hydroxyoctadecanoic acid was also identified. Limited studies have indicated a possible beneficial effect of di-hydroxyoctadecanoic acid on metabolism [26]. However, its biological relevance in the present study remains unclear. The aforementioned compounds are derivatives or oxidation products of oleic acid [27]. Moreover, (9Z,12E)-15,16-Dihydroxyoctadeca-9,12-dienoic acid (15,16-DiHODE), which is produced during the oxylipin pathway, was found at several time points after carob consumption. In general, hydroxy-octadecadienoic acids (HODEs) are known to be involved in processes related to early atherogenesis by activating PPARs, thereby influencing adipogenesis, lipid metabolism, and insulin signaling. In the production of 15,16-DiHODE, α-linolenic acid (ALA) is initially oxidized by CYP enzymes to form the epoxide 15,16-epODE, which is subsequently hydrolyzed by soluble epoxide hydrolase (sEH) to yield the diol 15,16-DiHODE [28,29,30]. Higher levels of such diols, either produced endogenously or received by diet, may enhance insulin resistance or a pro-inflammatory profile [31,32]. This metabolite was detected at different time points, which may suggest that intermittent or renewed in vivo production occurs, possibly via the previously described metabolic pathway. In contrast to the two linoleic acid derivatives, (12Z)-9,10-dihydroxyoctadec-12-enoic acid (9,10-DiHOME), an epoxide hydration product, did not follow the excretion patterns of its related compounds.

L-β-Homotyrosine, also present in pods, is a derivative of tyrosine, an amino acid present in carobs [33], consistent with the hypothesis of a potential metabolic conversion or a metabolic response post-consumption.

Gentiatibetine, palmitic acid, monosaccharides, myoinositol and γ–valerolactone were detected following carob consumption. Simple carbohydrates and carbohydrate conjugates, which are naturally present in Imera [8], also occur after consumption. Analyses of *Ceratonia siliqua* report palmitic acid as one of the main saturated fatty acids [34,35]. Particularly in carob pods, palmitic acid has been found to be the most abundant in the methanolic extract [8].

In plant sources, aconitic acid occurs predominantly in its trans isomer, which is considered the most stable form and is widely reported as the dominant isomer in plant matrices [36,37]. Trans-aconitate was detected here after consumption. Our previous study [7] reported the presence of this compound in the pods. Trans-aconitate is also a well-established inhibitor of aconitase, an enzyme known to exhibit reduced activity under oxidative conditions [38]. Overall, the observed postprandial appearance of trans-aconitate in serum may reflect short-term metabolic responses following intake; p-Cresol sulfate found in serum samples is considered a colon-derived metabolite, produced through bacterial metabolism of tyrosine, which may indicate a contribution of gut microbial activity to the observed changes [39].

In our study, it was anticipated that p-Cresol sulfate production would decrease after the consumption of the beverage, as high fiber content reduces the amount of amino acids that are fermented by gut microbiota. Also, the reduced colon transit expected in high fiber intake would delay amino acid conversion [39]. This discrepancy may be due to the fact that the 5-day wash-out period of a low-phytochemical diet was relatively high in protein, which may have contributed to a prolonged excretion kinetic of this metabolite.

Decrease in phe-phe levels postprandially may reflect peptide breakdown and potentially enhanced amino acid digestion and absorption facilitated by dietary fibers [40].

Among the detected compounds, enterolactone, urolithin B, and γ-valerolactone were classified as putative gut microbiota-related metabolites based on previous reports describing their microbial origin from dietary phenolic or related precursors [39,41,42,43]. Enterolactone, increased after the intake of carob beverage, is considered a gut-derived metabolite formed through the microbial conversion of plant lignans and related phenolic compounds [39]. Its detection in serum following carob consumption may suggest an intestinal fermentation of lignan-like precursors or structurally similar polyphenols naturally present in carobs [16]. Enterolactone has been associated with antioxidant, anti-inflammatory, and estrogen-modulating effects and may contribute to metabolic homeostasis through the regulation of glucose and lipid metabolism [44]. Therefore, its appearance post-carob ingestion could potentially involve a contribution of the gut microbiota to the systemic pool of phenolic metabolites, although its specific role in the present study cannot be established. Additionally, urolithin B reflects a microbial metabolite resulting from the transformation of ellagic acid and ellagitannins [42,43]. Although carob pods do not contain ellagitannins, their phenolic acids and condensed tannins may possess structural features enabling analogous microbial metabolism, potentially giving rise to urolithin derivatives [2,16]. Urolithins are reported to be associated with anti-inflammatory and antioxidant effects and promote mitochondrial function through the activation of mitophagy [43].

The kinetics in antioxidant capacity were included to provide complementary information on systemic redox following carob intake. While the comparisons in antioxidant potential did not survive the Bonferroni adjustment, a significant main effect was evident in lag time. Serum resistance to oxidation after phenolic intake does not always parallel serum phenolic peaks, likely because the effect involves mechanisms beyond circulating phenolic concentration. For example, a postprandial intake of phenol-rich extra-virgin olive oil reduced oxidized low-density lipoprotein and upregulated antioxidant-related genes (i.e., catalase and SOD1) without a rise in serum phenolics [44]. Later secondary changes in serum antioxidant capacity beyond 3 h cannot be excluded. While serum metabolite profiling was monitored for 6 h in order to cover both the absorption and elimination period, antioxidant capacity was not extended further than 3 h in order to examine only the direct effects associated with the consumption of the carob beverage and avoid confounding the effect of gut microbiome-derived metabolites. This is further confirmed by the fact that microbial metabolites, like γ-valerolactone formed from flavan-3-ol metabolism, were detected at 3 h post-consumption and retain or even exceed the antioxidant and anti-inflammatory properties of the parent flavonoids [45].

We acknowledge that the lack of a placebo control group and the single-arm design of this acute study should be considered when interpreting the findings. However, this exploratory work serves as a proof-of-concept time-course analysis, mapping for the first time the dynamic postprandial appearance of metabolites in human circulation after the intake of a carob pod preparation. Although a control beverage would further strengthen causal interpretation, the longitudinal design, in which participants served as their own baseline, together with strict inclusion and exclusion criteria and successful dietary washout, increased the robustness of the findings. The inclusion of only male participants reduced biological variability related to hormonal fluctuations, although future studies including females are needed to clarify possible sex-related differences. The determination of only reducing and not eliminating antiradical activity in serum may be considered an additional limitation. Although the detection of enterolactone, urolithin B, and γ-valerolactone may suggest a contribution of gut microbial metabolism, the current untargeted approach does not allow for direct confirmation that these metabolites were generated from carob phenolics, and further targeted analyses, preferably combined with a gut microbiota assessment, are needed to confirm this pathway. Yet, the untargeted LC-TOF-MS approach enabled broad metabolite mapping and exploration of the metabolic pathways that may be influenced by carob consumption.

## 5. Conclusions

This novel study demonstrates that the use of a highly sensitive LC-TOF-MS platform enabled, for the first time, the identification of twenty-four metabolites in the serum of healthy male participants following consumption of a carob beverage prepared from pods. These metabolites included parent compounds, related metabolites, and additional endogenous serum metabolites, comprising phenolics, fatty acids, amino acids, dipeptides, monosaccharides, pyridoxine, and γ-valerolactone. Changes in serum antioxidant capacity, assessed by copper sulfate-induced serum oxidation and FRAP assays, did not parallel the kinetic patterns of the phenolic compounds detected in this study. This suggests that the antioxidant response may be influenced by mechanisms beyond circulating phenolics, including the contribution of endogenous antioxidants.

Future studies using a placebo-controlled design and targeted metabolite analysis are needed to confirm the identified compounds and further clarify the metabolic pathways involved. In addition, studies including female participants are warranted to examine potential sex-related differences.

## Figures and Tables

**Table 1:** Inclusion and exclusion criteria for participation in the study.

Inclusion Criteria
Adult, male subjects (≥18 years of age), healthy status, normal body weight (as defined by normal body fat percentage)
Exclusion Criteria
Occupation of dietitian/nutritionist and/or nutrition-related studies, type 1 diabetes, cardiovascular diseases, hypertension, thyroid disorder, liver disease, kidney disease, gastrointestinal disease, mental illness, use of nutraceutical supplements or natural products for weight loss, high body fat mass

**Table 2:** Example of one-day low-phytochemical diet.

Meal	Description
Breakfast	240 mL of milk, or 180 g yogurt with 1 cup of cereal, and 1 boiled egg, or 1 toast with cheese and deli meat.
Snack	1 Thessaloniki bagel (koulouri) and 1 slice of cheese or 1 toast with cheese.
Lunch	180 g of meat (steak, chicken, fillet, or burger patty) with 2 cups of pasta, or rice, or potatoes, and 1 tablespoon of olive oil (either cooked or raw).
Dinner	120 g of meat (steak, chicken, fillet, or burger patty) with 2 cups of pasta, or rice, or potatoes, and 1 tablespoon of olive oil (either cooked or raw).

**Table 3:** Baseline characteristics of the sample.

Subjects Characteristics	Mean ± SD
Age (years)	24.47 ± 4.82
Height (cm)	1.78 ± 0.05
Weight (kg)	76.85 ± 6.63
BMI (kg/m^2^)	24.65 ± 1.79
Fat (%)	15.35 ± 3.87
Free fat mass (kg)	64.96 ± 5.15
Total body water (kg)	47.55 ± 3.79
Systolic pressure (mg/Hg)	126.13 ± 8.25
Diastolic pressure (mm/Hg)	68.88 ± 7.47
Pulse rate (beats/minute)	67.13 ± 11.53

BMI: Body mass index.

**Table 4:** MS-based identification features of the tentative metabolites and their time-dependent occurrence.

A/A	Putative Metabolite	Chemical Class	MS ((M − H)^−^ or (M + H)^+^)/MS2 Fragments	RT (min)	Peak Intensity (×10^2^) (a.u) (mean ± SD)
1	(−) Citramalic acid	Carboxylic acids and derivatives	146.9452 (M − H)^−^/146.8911, 103.0296, 87.1282	6.05	2.30 (±0.87) _t = 30_
2	(9Z,12E)-15,16-Dihydroxyoctadeca-9,12-dienoic acid (15,16-DiHODE)	Fatty acyls	311.2214 (M − H)^−^/223.1560. 183.1013	8.42	6.02 (±0.60) _t = 0_, 13.1 (±1.1) _t = 30_, 11.0 (±3.3) _t = 120_, 12.3(±3.1) _t = 240_
3	(12Z)-9,10-Dihydroxyoctadec-12-enoic acid (9,10-DiHOME)	Oxylipins	313.1923 (M − H)^−^/295.1025, 277.1316	7.81	86.7(±8.1), _t = 150_
4	9-Hydroxy-10,12-octadecadienoic acid (9-HODE)	Fatty acyls	295.2188 (M − H)^−^/277.2095, 249.1712, 171.1024	9.70	12.7 (±1.5) _t = 0_, 13.7 (±1.5) _t = 30_, 14.0 (±1.0) _t = 60_, 11.16 (±0.8) _t = 90_, 13.7 (±2.5) _t = 120_, 11.3 (±2.4) _t = 150_, 11.2 (±2.6) _t = 180_, 11.30 (±0.6), _t = 240_, 11.3 (±1.6) _t = 300_, 11.6 (±1.8) _t = 360_
5	Benzoic acid	Benzoic acids and derivatives	121.0335 (M − H)^−^/93.0418, 77.0374	5.77	0.91(±0.14) _t = 60_
6	Citric acid	Carboxylic acids and derivatives	190.9269 (M − H)^−^/120.0414, 115.0065, 102.9518, 87.9296	6.23	10.0 (±2.0) _t = 60_, 11.0 (±3.0) _t = 120_, 9.50 (±0.6) _t = 180_, 9.0 (±2.0) _t = 240_, 10.4 (±1.8) _t = 300_, 10.60 (±0.89) _t = 360_
7	Coumaroyl hexoside	Phenolic Glycoside	325.1936 (M − H)^−^/183.0168, 163.0318, 144.9650, 119.0543	8.63	42.9 (±2.6) _t = 0_, 48.1 (±3.2) _t = 30_
8	Di-hydroxyoctadecanoic acid	Hydroxy fatty acids	315.2576 (M − H)^−^/297.2356, 279.2679, 171.1105, 155.1089, 141.0355, 127.0117	8.52	40.1 (±1.8) _t = 0_, 43.9 (±3.9) _t = 30_, 41.2 (±3.3) _t = 60_, 69.2 (±5.4) _t = 90_, 44.5 (±1.9) _t = 120_, 43.3 (±3.5) _t = 150_, 42.4 (±1.9) _t = 180_, 42.9 (±1.8) _t = 240_, 42.6 (±2.0) _t = 300_, 41.5 (±1.1) _t = 360_
9	Enterolactone	Lignans, related compounds	297.125 (M − H)^−^/253.1420, 121.1350	8.52	17.3 (±4.5) _t = 0_, 151 (±14) _t = 30_, 67.7 (±8.5) _t = 90_
10	Gentiatibetine	Alkaloids	166.0864 (M + H)^+^/151.0725, 120.0811	10.17	989 (±11) _t = 30_, 1133 (±126) _t = 60_
11	Hydroxylated oleic acid	Hydroxy Fatty Acid	297.2426 (M − H)^−^/277.2099, 171.1028	8.32	21.7 (±1.7) _t = 60_, 23.4 (±1.8) _t = 90_
12	Hydroxyoctadecenedioic acid	Dicarboxylic acids	327.2162 (M − H)^−^/309.2054, 283.2173, 265.2097	6.78	50.3 (±2.1) _t = 30_, 61.0 (±3.0) _t = 60_, 63.0 (±3.0) _t = 90_, 87.0 (±5.0) _t = 120_, 79.3 (±4.0) _t = 150_, 63.6 (±3.5) _t = 180_, 61.7 (±4.2) _t = 240_, 65.3 (±3.1) _t = 300_, 65.6 (±4.5) _t = 360_
13	L-beta-Homotyrosine	Amino Acids	194.0811 (M − H)^−^/150.0166, 133.0700	8.05	32.6 (±3.5) _t = 0_, 36.7 (±3.5) _t = 60_, 23.8 (±1.6) _t = 90_, 19.3 (±1.5) _t = 120_, 16.0 (±2.0) _t = 240_, 17.8 (±2.7) _t = 300_
14	L-Tryptophan	Amino Acids	205.0982 (M + H)^+^/146.0609, 132.0809, 118.0660	11.24	6133 (±351) _t = 0_, 6300 (±300) _t = 30_
15	Luteolin 7-methyl ether		301.0809 (M + H)^+^/286.0463, 153.1022	11.83	119.3 (±8.0) _t = 30_
16	Monosaccharides (fructose or mannose or sorbose) or myoinositol	Carbohydrates, carbohydrate conjugates	179.0562 (M − H)^−^/161.0991, 131.0640, 101,0515, 89.0324	1.28	3.43 (±0.47) _t = 30_, 2.67 (±0.45) _t = 60_
17	p-Coumaric acid	Phenylpropanoids, polyketides	163.0468 (M − H)^−^/119.0510, 93.0357	1.60	3.3 (±1.2) _t = 60_
18	p-Cresol sulfate	Phenolic sulfates	187.0060 (M − H)^−^/107.0550, 80.9476	9.63	53.3 (±6.5) _t = 0_, 130.0 (±7.0) _t = 30_, 58.4 (±1.6) _t = 120_, 127.5 (±3.3) _t = 150_
19	Palmitic acid	Fatty acyls	255.2333 (M − H)^−^/239.2371, 211.2523, 203.2024	2.48	2.13 (±0.50) _t = 30_, 12.9 (±4.1) _t = 90_, 3.70 (±0.30) _t = 150_, 2.50 (±0.50) _t = 240_
20	Phe-Phe	Dipeptide	313.1573 (M + H)^+^/120.0815, 103.0542	11.10	459.7 (±6.5) _t = 0_, 379.8 (±4.5) _t = 30_, 380.5 (±2.1) _t = 60_
21	Pyridoxine	Vitamins	170.0841 (M + H)^+^/152.0645, 134.0614	1.77	2.30 (±0.50) _t = 60_
22	trans-Aconitate	Carboxylic acids, derivatives	173.0093 (M − H)^−^/129.0179, 111.0880, 85.0565	5.35	7.1 (±1.9) _t = 30_
23	Urolithin B	Benzenoids, phenolic metabolites	213.0342 (M + H)^+^/169.0836, 139.0065	11.74	28.0 (±4.0) _t = 30_
24	γ-Valerolactone	Lactones	101.1202 (M + H)^+^/83.0602	12.56	0.147 (±0.04) _t = 180_, 0.183 (±0.03) _t = 240_

**Table 5:** Mean levels of antioxidant capacity at each time point.

	BASELINE	30 min	60 min	90 min	120 min	150 min	180 min	*p*-Value
FRAP (nmol/well)	15.5 ± 3.8	13.6 ± 4.4	15.0 ± 2.4	14.0 ± 2.7	14.7 ± 5.4	16.7 ± 4.7	14.6 ± 4.1	0.332
Lag time (s)	7173.8 ± 1518.8	7913.9 ± 2016.7	6713.0 ± 1050.0	7784. 8 ± 1702.6	7523.0 ± 1765.3	7551.2 ± 1687.9	9093.5 ± 1885.1	0.001 *

The data are expressed as mean ± standard deviation. Differences between time points were evaluated with repeated measurements ANOVA after Bonferroni correction (*n* = 15). FRAP: ferric-reducing antioxidant power. Level of significance was set as 0.05. * Although a significant time effect was observed for Lag time (*p* = 0.001), pairwise comparisons between different time points after Bonferroni correction did not identify any significant changes, suggesting a potential trend rather than a distinct change.

## Data Availability

The data presented in this study are available on request from the corresponding author due to (specify the reason for the restriction).

## Supplementary Materials

The following supporting information can be downloaded at: https://www.mdpi.com/article/10.3390/nu18132190/s1, Figure S1a. MS/MS fragments of coumaroyl-hexoside at negative ionization. Figure S1b. MS/MS fragments of Di-hydroxyoctadecanoic acid at negative ionization. Figure S1c. MS/MS fragments of p-Cresol sulfate at negative ionization. Figure S1d. MS/MS fragments of Phe-Phe at positive ionization.

## Abbreviations

The following abbreviations are used in this manuscript:

ACN	Acetonitrile
ALA	Alpha-Linolenic Acid
ANOVA	Analysis of Variance
BMI	Body Mass Index
CE	Collision Energy
CUR	Curtain Gas
CYP	Cytochrome P450
DP	Declustering Potential
ESI	Electrospray Ionization
FRAP	Ferric-Reducing Antioxidant Power
GAS1	Ion Source Gas 1
GAS2	Ion Source Gas 2
HMDB	Human Metabolome Database
HODE	Hydroxyoctadecadienoic Acid
IDA	Information-Dependent Acquisition
LC-MS	Liquid Chromatography–Mass Spectrometry
LC-TOF-MS	Liquid Chromatography–Time-of-Flight Mass Spectrometry
LSD	Least Significant Difference
MS	Mass Spectrometry
MS/MS	Tandem Mass Spectrometry
QC	Quality Control
ROS	Reactive Oxygen Species
RP	Reversed Phase
RT	Retention Time
SD	Standard Deviation
sEH	Soluble Epoxide Hydrolase
SOD1	Superoxide Dismutase 1
SPSS	Statistical Package for the Social Sciences
TCA	Tricarboxylic Acid
TTOF-MS	Triple-Quadrupole Time-of-Flight Mass Spectrometry
UPLC-Q-TOF-MS	Ultra-Performance Liquid Chromatography–Quadrupole Time-of-Flight Mass Spectrometry
WC	Waist Circumference
WHO	World Health Organization
