# Supplementary material for: Postprandial Metabolite and Antioxidant Kinetics Following Intake of a Carob Beverage in Healthy Males
Source: Nutrients. 2026 Jul 5;18(13):2190. doi: 10.3390/nu18132190 (PMC13364267; doi:10.3390/nu18132190)

Supplementary material relative to

Postprandial Metabolite and Antioxidant Kinetics Following Intake of a Carob Beverage in Healthy Males

Stamatia-Angeliki Kleftaki [1,†], Thalia Tsiaka [2,†], Charalampia Amerikanou [1], Demetra Sigala [1], Aikaterini Mavroudi [1], Maria-Myrto Karagiorgou [1], Altenisa Kuci [1], Chara Tzavara [1], Vasiliki Dima [2], Maria Morfiadaki [2], Aristea Gioxari [1], Panagiotis Zoumpoulakis [2] and Andriana C. Kaliora [1,*]

[1] Department of Nutrition and Dietetics, School of Health Science and Education, Harokopio University of Athens, 70 El. Venizelou Ave., 17676 Athens, Greece; matina.kleftaki@gmail.com (S.-A.K.); amerikanou@windowslive.com (C.A.); sigalademetra@gmail.com (D.S.); caterina@mavroudis.gr (A.M.); marymkar@gmail.com (M.-M.K.); koutsialtenisa@gmail.com (A.K.); htzavara@med.uoa.gr (C.T.); a.gioxari@go.uop.gr (A.G.)

[2] Department of Food Science and Technology, University of West Attica, Agiou Spyridonos, 12243 Egaleo, Greece; tsiakath@uniwa.gr (T.T.); fst20684149@uniwa.gr (V.D.); fst19684059@uniwa.gr (M.M.); pzoump@uniwa.gr (P.Z.)

* Correspondence: akaliora@hua.gr

† These authors contributed equally to this work.

Figure S1a. MS/MS fragments of coumaroyl-hexoside at negative ionization.

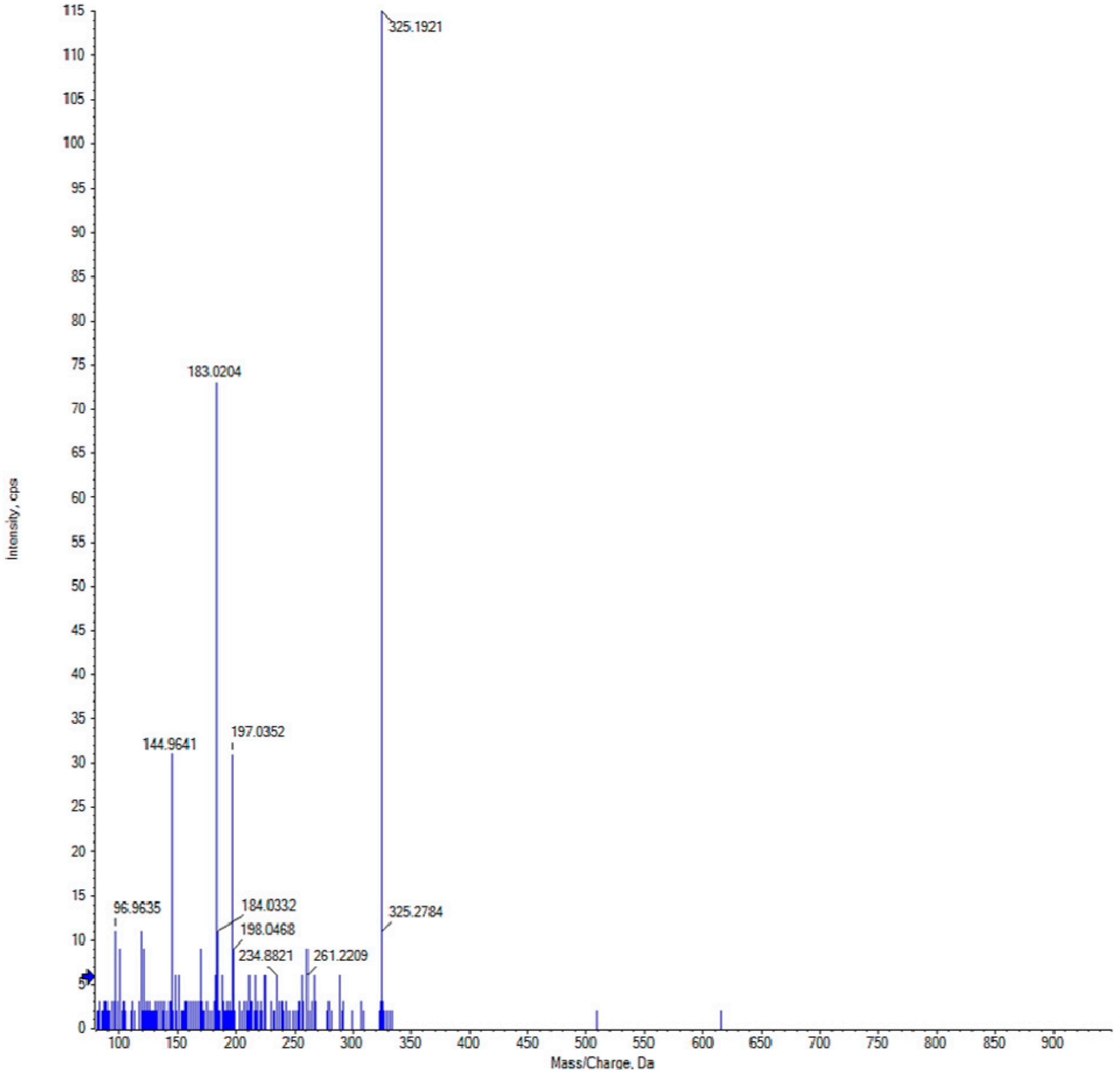

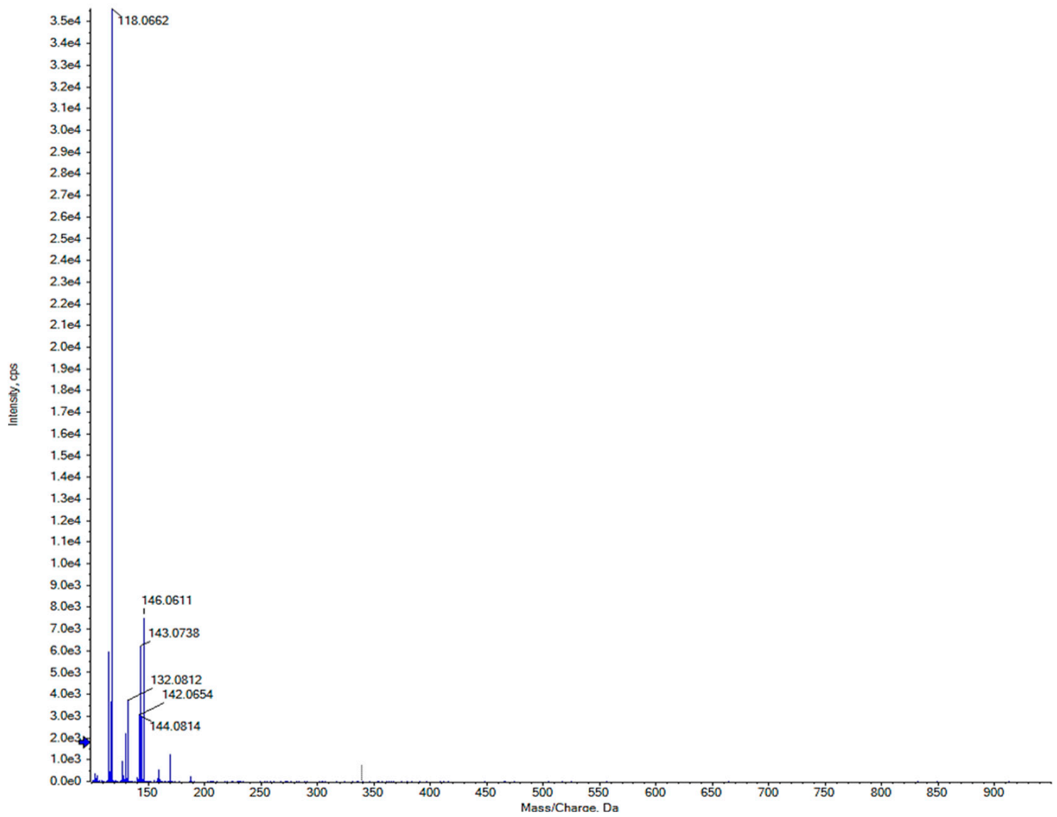

118.0662
146.0611
143.0738
132.0812
142.0654
144.0814
Intensity, cps
Mass/Charge, Da

Figure S1b. MS/MS fragments of Di-hydroxyoctadecanoic acid at negative ionization.

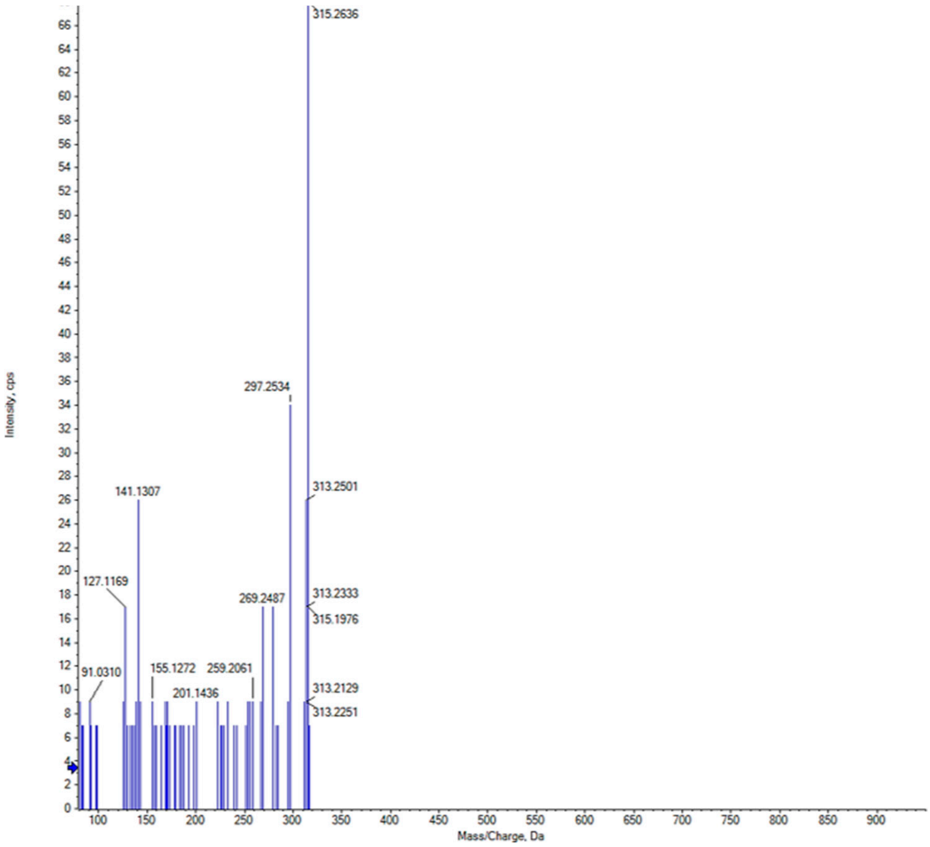

Figure S1c. MS/MS fragments of p-Cresol sulfate at negative ionization.

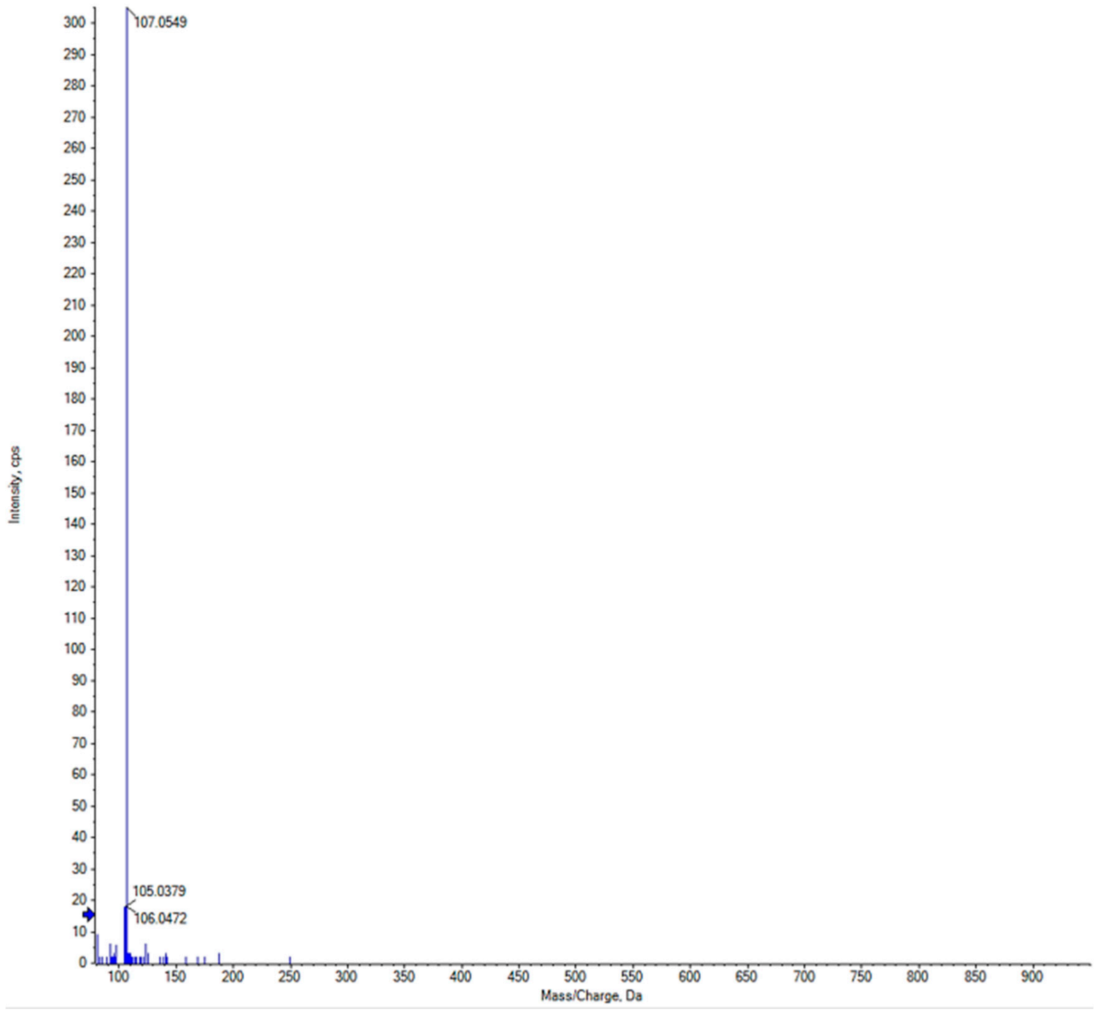

Figure S1d. MS/MS fragments of Phe-Phe at positive ionization.

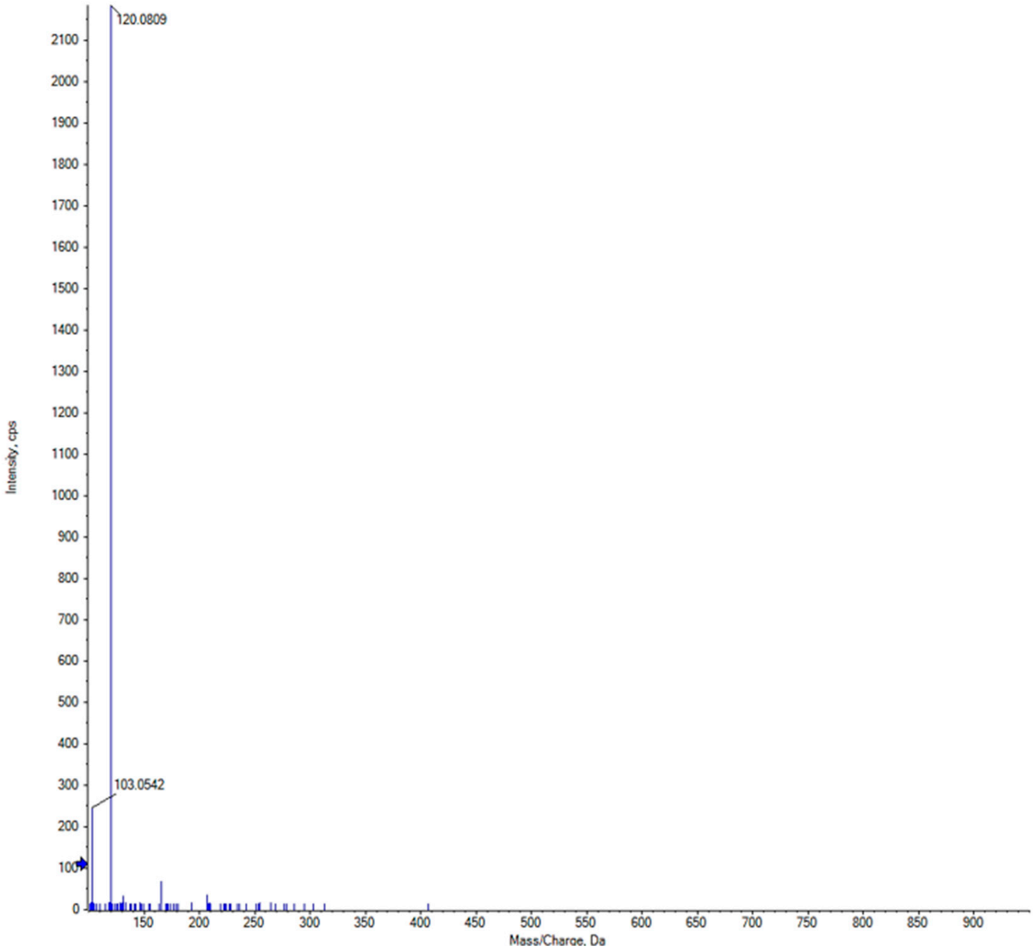

Supplement: Supplementary file 1 [file nutrients-18-02190-s001.zip › nutrients-4368712-supplementary.pdf]
